# Zinc-α-2-Glycoprotein: A Candidate Biomarker for Colon Cancer Diagnosis in Chinese Population

**DOI:** 10.3390/ijms16010691

**Published:** 2014-12-30

**Authors:** Yingming Xue, Fudong Yu, Dongwang Yan, Feifei Cui, Huamei Tang, Xiaoliang Wang, Jian Chen, Huijun Lu, Senlin Zhao, Zhihai Peng

**Affiliations:** 1 Department of General Surgery, Shanghai Jiao Tong University Affiliated First People’s Hospital, Shanghai 200000, China; E-Mails: sjtuyingmingxue@163.com (Y.X.); fudanyu@yeah.net (F.Y.); yandw70@163.com (D.Y.); vivicuiff@163.com (F.C.); xiaoliangwang1975@163.com (X.W.); yufuchenjian@gmail.com (J.C.); senlinzhao1989sh@163.com (S.Z.); 2 Department of Pathology, Shanghai Jiao Tong University Affiliated First People’s Hospital, Shanghai 200000, China; E-Mails: tanghuamei@gmail.com (H.T.); luhuijun88885@163.com (H.L.)

**Keywords:** AZGP1 protein, human, colon cancer, clinical marker, tissue array analysis

## Abstract

Zinc-α-2-glycoprotein (AZGP1) is a 41-kDa secreted glycoprotein, which has been detected in several malignancies. The diagnostic value of AZGP1 in serum of prostate and breast cancer patients has been reported. Analyzing “The Cancer Genome Atlas” data, we found that in colon cancer *AZGP1* gene expression was upregulated at transcriptional level. We hypothesized that AZGP1 could be used as a diagnostic marker of colon cancer. First, we confirmed AZGP1 expression was higher in a set of 28 tumor tissues than in normal colonic mucosa tissues by real-time quantitative PCR and western blot in a Chinese population. We verified that serum concentration of AZGP1 was higher in 120 colon cancer patients compared with 40 healthy controls by ELISA (*p* < 0.001). Then receiver-operating characteristic (ROC) curve analysis was used to evaluate the predictive diagnostic value of AZGP1 in serum. The area under the curve (AUC) of AZGP1 was 0.742 (*p* < 0.001, 95% confidence interval (*CI*) = 0.656–0.827) in between the AUC of carcinoembryonic antigen (CEA) and the AUC of CA19-9, suggesting that predictive diagnostic value of AZGP1 is between CEA and Carbohydrate 19-9 (CA19-9). The combination of AZGP1 with traditional serum biomarkers, CEA and CA19-9, could result in better diagnostic results. To further validate the diagnostic value of AZGP1, a tissue microarray containing 190 samples of primary colon cancer tissue paired with normal colonic tissue was analysed and the result showed that AZGP1 was significantly upregulated in 68.4% (130 of 190) of the primary cancer lesions. In contrast, there was a weakly positive staining in 29.5% (56 of 190) of the normal colonic tissue samples (*p* < 0.001). Leave-one-out cross-validation was performed on the serum data, and showed that the diagnostic value of AZGP1 had 63.3% sensitivity and 65.0% specificity. Combination of AZGP1, CEA and CA19-9 had improved diagnosis value accuracy with 74.2% sensitivity and 72.5% specificity. These results suggest that AZGP1 is a useful diagnostic biomarker in tissues and serum from a Chinese population.

## 1. Introduction

Colon cancer has one of the highest incidence rates among different types of cancers worldwide and remains the second most common cause of cancer-related death in the United States [1,2]. In the past few years, surgery, adjuvent chemotherapy and individualized molecular targeted therapy administered to patients significantly improved the survival rate, with the five-year overall survival rate at 93%, 83%, 60% and 8% in stage I, II, III and IV, respectively [3]. Undeniably, the five-year survival rate for metastatic colorectal cancer is still approximately less than 10% [4]. In China and other developing countries, due to changes in lifestyle and dietary habits, the incidence of colon cancer has rapidly increased to become the second leading cause of death due to cancer [5,6]. A proportion of early stage patients receiving surgical resection still relapse, even with combined treatment of adjuvent chemotherapy [3]. Many markers such as adenomatous polyposis coli (APC), DNA mismatch repair genes, vascular endothelial growth factors (VEGFs) and their receptors, and, more recently, microRNAs and colon epithelial stem cell markers, have been systematically analyzed for diagnosis and prognosis of colon cancer [7,8,9,10,11,12]. Currently, carcinoembryonic antigen (CEA) and Carbohydrate 19-9 (CA19-9) are still the most commonly used clinical biomarkers, but their accuracy does not meet clinical needs. This highlights the need for new biomarkers for a more precise prediction of tumorigenesis and/or recurrence and consequently improved individualised therapeutic regimens after surgery.

The colon and rectum originate from different embryological tissue, and relevant studies have demonstrated that they have different physiological functions, histochemical reactions and gene expression profiles [13,14]; we decided to focus our research, and chose to further investigate colon cancer.

Zinc-α-2-glycoprotein (AZGP1), a 41-kDa secreted glycoprotein, has been detected in several malignancies including breast, prostate, and lung cancer [15,16,17,18]. AZGP1 was found to be a potential biomarker for prostate cancer and could be used for early diagnosis [19]. In prostate cancer, AZGP1 also showed significant diagnostic value as a serum marker [20]. We hypothesized that AZGP1 could be used as a diagnostic marker of colon cancer.

To prove this hypothesis, we examined AZGP1 differential expression in colon cancer specimens and paired normal mucosa specimens by using quantitative real time polymerase chain reaction (PCR) and western blot (WB). We also detected the difference in serum of cancer patients and normal controls by Enzyme-linked immunosorbent assay (ELISA), and made Receiver operating characteristic (ROC) curves to explore the diagnostic value of AZGP1 either alone or combined with the common clinical biomarkers of CEA and CA19-9. To further validate whether AZGP1 could be used as a general detection biomarker, verification was carried out by using immunohistochemical staining in tissue microarray (TMA) composed of large samples and leave-one-out method analysis in serum data. Here, we identified AZGP1 as a potential biomarker for colon cancer.

## 2. Results and Discussion

### 2.1. AZGP1 Expression in Colon Cancer at Transcriptional Level

**Figure 1:**
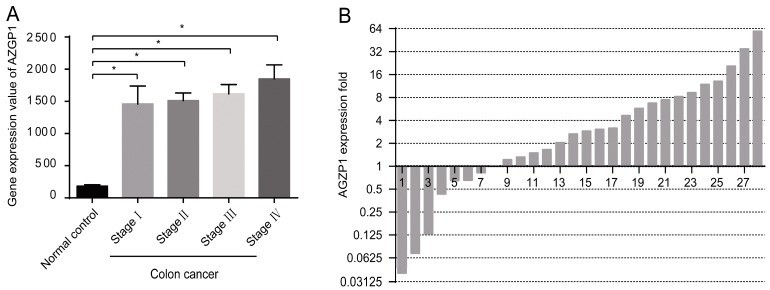
*AZGP1* expression in colon cancer at the transcriptional level. (**A**) Gene expression of *AZGP1* in colon cancer samples from TCGA data. The y-coordinate represents *AZGP1* gene expression value among 236 colon cancer samples with HiSeq sequencing expression value. * *p* < 0.005 compared with control group. The results are presented as mean ± SE; (**B**) Relative expression of *AZGP1* gene in a series of 28 matched colon cancerous tissue specimens compared with that in normal mucosa specimens from a Chinese population. A logarithmic scale of 2^−ΔΔ*C*t^ is used to represent the fold change in quantitative real-time PCR detection.

After analysis of the “The Cancer Genome Atlas” (TCGA) data, we found that in colon cancer *AZGP1* gene expression was upregulated at the transcriptional level (Figure 1A) (*p* < 0.005). Further, we detected mRNA expression by qPCR in a set of 28 tumor tissues and paired normal colonic mucosa tissues; 16 colon cancer tissue samples (57.1%) exhibited high expression of AZGP1 mRNA, showing at least a two-fold increase, when compared to expression of AZGP1 mRNA in paired normal colonic mucosa tissue samples (Figure 1B). Our data showed that AZGP1 expression was upregulated in colon cancer patients at the transcriptional level in the Chinese population (*p* = 0.015) (Figure S1) in accordance with the TCGA data. The above results suggested that the AZGP1 transcript level might be used as a diagnostic marker for colon cancer.

### 2.2. AZGP1 Expression in Colon Cancer Confirmed at the Posttranscriptional Level

**Figure 2:**
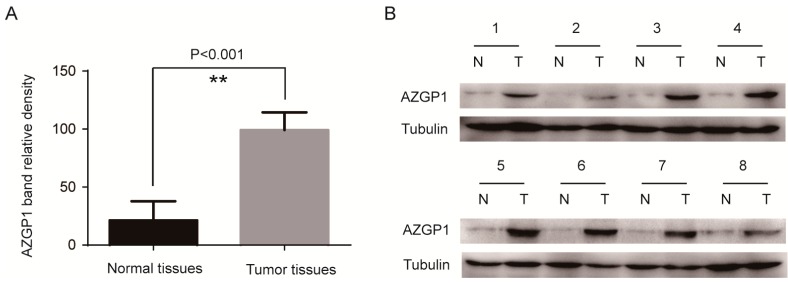
Upregulation of AZGP1 expression in colon cancer at the posttranscriptional level. Western blotting analysis of AZGP1 protein expression in 28 matched colon cancerous tissue specimens compared with that in normal mucosa specimens. (**A**) The relative density value of AZGP1 protein bands derived from normal mucosa specimens and colon cancerous tissue specimens, respectively (*p* < 0.001); (**B**) Western blotting analysis of AZGP1 protein expression in representative 8 paired colon tumor tissues. Tubulin was used as the loading control. ** *p* < 0.001, N represents normal mucosa specimen and T represents tumor tissue specimen.

AZGP1 protein expression was evaluated by western blotting in the above-mentioned samples (Figure 2); the relative density value of normal colonic mucosa bands was 21.36 ± 16.42, and the relative density value of colon cancer bands was 99.14 ± 15.26. The AZGP1 protein expression in colon cancer was significantly higher than in normal colonic mucosa (Figure 2A) (*p* < 0.001). These results indicate that AZGP1 expression was also upregulated in colon cancer patients in the Chinese population at the posttranscriptional levels, confirming that the *AZGP1* gene also could be used as a diagnosistic marker at the protein level.

### 2.3. AZGP1 Concentration in Serum and Its Diagnostic Value for Colon Cancer

**Figure 3:**
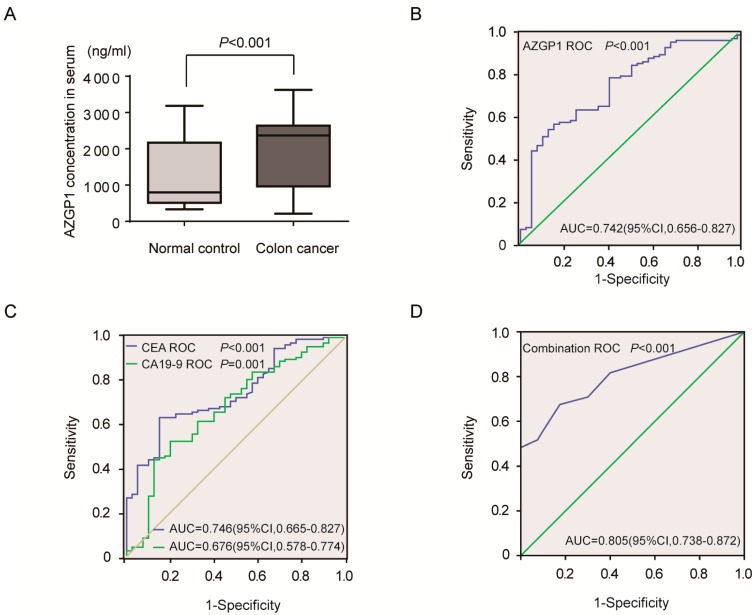
High serum concentrations of AZGP1 in colon cancer patients and high diagnostic value of serum AZGP1 for colon cancer. (**A**) Serum concentration of AZGP1 in colon cancer patients is higher compared with that in healthy controls (*p* < 0.001); (**B**) The ROC of AZGP1. The AUC is 0.742 (*p* < 0.001, 95% *CI* = 0.656–0.827). At the cutoff value of 2297.71 ng/mL for AZGP1, the sensitivity is 55.8% and the specificity is 85.0%; (**C**) The ROC of CEA and CA19-9. The AUC of CEA and CA19-9 are 0.746 (*p* < 0.001, 95% *CI* = 0.665–0.827) and 0.676 (*p* = 0.001, 95% *CI* = 0.578–0.774). At the cutoff value of 6.47 ng/mL for CEA and 44.77 U/mL for CA19-9, the sensitivity and specificity are 63.3% and 85%, 52.5% and 80.0%, respectively; (**D**) The ROC of combination of AZGP1, CEA and CA199. The AUC is 0.805 (*p* < 0.001, 95% *CI* = 0.738–0.872). With a cutoff value of 2.13, the sensitivity and specificity are 67.5% and 82.5%, respectively.

AZGP1 is a secreted protein, and we thus analyzed its serum level by ELISA. The results showed that serum level of AZGP1 in 120 colon cancer patients was higher compared with 40 healthy controls (*p* < 0.001) (Figure 3A). To further explore the clinical significance of the serum level of AZGP1, we detected the association of AZGP1 serum level with patient clinicopathological parameters and found that AZGP1 serum level was positively associated with American Joint Committee on Cancer (AJCC) clinical stage (*p* < 0.001), T classification (*p* < 0.001), lymph node metastasis (*p* < 0.001) and distant metastasis (*p* < 0.001). (Table S1). To explore the diagnostic value of serum AZGP1, we also measured the serum concentration of conventional serum tumor markers, CEA and CA19-9, by ELISA method. The receiver-operating characteristic (ROC) curve analyses revealed that serum level of AZGP1 was a practical diagnostic biomarker for differentiating colon cancer patients from controls within ROC curve areas of 0.742 (*p* < 0.001, 95% confidence interval (*CI*) = 0.656–0.827). At the cutoff value of 2297.71 ng/mL for AZGP1, the sensitivity was 55.8% and the specificity was 85.0% (Figure 3B). The area under the curve (AUC) of CEA and CA19-9 were 0.746 (*p* < 0.001, 95% *CI* = 0.665–0.827) and 0.676 (*p* = 0.001, 95% *CI* = 0.578–0.774). At the cutoff value of 6.47 ng/mL for CEA and 44.77 U/mL for CA19-9, the sensitivity and specificity were 63.3% and 85%, 52.5% and 80.0%, respectively (Figure 3C). Multivariate logistic regression analyses, which included AZGP1, CEA and CA19-9, were used to combine three of them. The AUC of a combination of three markers was 0.805 (*p* < 0.001, 95% *CI* = 0.738–0.872). With a cutoff value of 2.13, the sensitivity and specificity were 67.5% and 82.5%, respectively (Figure 3D).

Our data provides independent statistically theoretical validation that the diagnostic value of AZGP1 was higher than CA19-9, and the diagnostic value of the combination was higher than each alone, suggesting that the combination could improve diagnostic accuracy.

### 2.4. AZGP1 Diagnostic Value for Colon Cancer Validated in TMA and Serum

**Figure 4:**
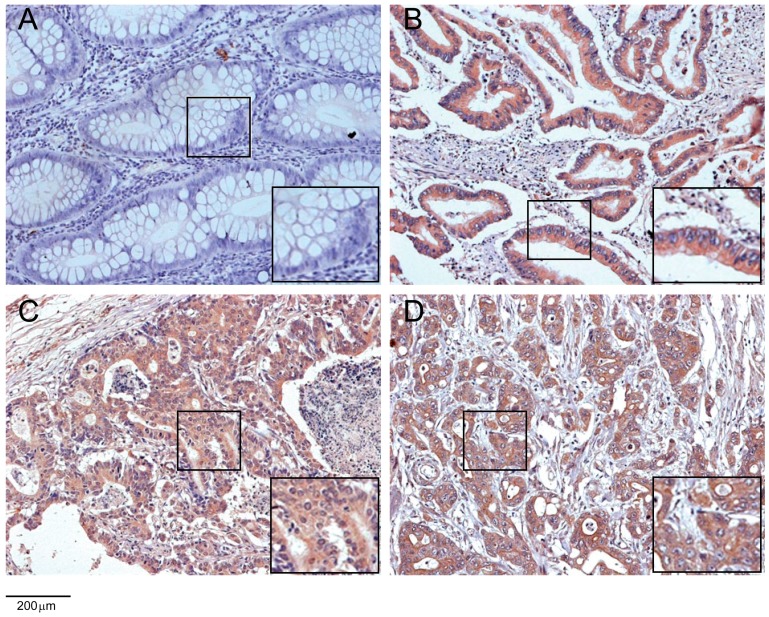
AZGP1 overexpression in colon tumorous tissues validated by TMA analysis.(**A**) Negative AZGP1 expression in normal colonic epithelium; (**B**) Strong AZGP1 staining in well-differentiated colon tumor tissues; (**C**) moderately differentiated colon tumor tissues and (**D**) poorly differentiated colon tumors tissues. Magnification ×200.

Tissue microarray containing 190 samples of primary colon cancer tissues paired with normal colonic tissues was used to determine AZGP1 expression by immunohistochemistry. As shown in Figure 4, AZGP1 protein was localized in the cytoplasm of cancer cells. Although AZGP1 protein was detected in the normal mucosa, the expression was very weak. AZGP1 was significantly upregulated in 68.4% (130 of 190) of the primary cancer lesions. In contrast, there was a weakly positive staining in 29.5% (56 of 190) of the normal colonic tissue samples (*p* < 0.001) (Figure 4). Additionally, we evaluated the association between AZGP1 expression and clinicopathological factors as summarized in Table 1. AZGP1 expression was positively associated with AJCC clinical stage (*p* = 0.024), T classification (*p* = 0.012), and lymph node metastasis (*p* = 0.005) (Table 1).

**Table 1:** Correlation between AZGP1 expression and clinicopathologic characteristics.

Case			AZGP1 Expression			*p*
			Negative	Weak	Strong	
Age	<66	75	21	28	25	0.649
	≥66	115	39	37	40	
Gender	Male	82	23	24	35	0.099
	Female	108	37	41	30	
Location	Right	78	25	26	27	0.965
	Transverse	16	5	6	5	
	Left	20	5	6	9	
	sigmoid	76	25	27	24	
T stage	T1	8	5	3	0	0.012
	T2	23	10	10	3	
	T3	71	26	18	27	
	T4	88	19	34	35	
N stage	N0	108	42	37	29	0.005
	N1	54	16	19	19	
	N2	28	2	9	17	
Metastasis	M0	173	58	57	58	0.175
	M1	17	2	8	7	
AJCC Stage	I	24	11	11	2	0.024
	II	81	30	25	26	
	III	68	17	21	30	
	IV	17	2	8	7	
Vessel invasion	No	179	59	62	58	0.083
	Yes	11	1	3	7	
Differentiation	Well	96	37	29	30	0.059
	Moderate	68	21	24	29	
	Poor	26	2	12	12	

To further examine the diagnostic value, leave-one-out cross-validation was performed on the serum data used above, and showed that the AZGP1 diagnostic value had 63.3% sensitivity and 65.0% specificity and was more sensitive than CA19-9; the combination of AZGP1, CEA and CA19-9 had a more accurate diagnosis value with 74.2% sensitivity and 72.5% specificity (Table 2). These results demonstrate that AZGP1 in tissues and serum was a useful diagnostic biomarker.

**Table 2:** Validating the diagnostic value of AZGP1, CEA and CA19-9 and the combination by leave-one-out cross-validation.

Serum Biomarkers	Sensitivity	Specificity	Accuracy
AZGP1	63.3	65.0	63.7
CEA	65.0	75.0	67.5
CA19-9	60.8	67.5	62.5
CEA + CA19-9	67.5	70.0	68.1
AZGP1 + CEA + CA19-9	74.2	72.5	73.8

## 3. Discussion

Early in 1961, Burgi *et al*. [21] separated and purified AZGP1 protein from human plasma and explored its chemical structure and chemical properties. AZGP1 is a 41-kDa secreted glycoprotein with a tendency to precipitate with zinc salts and with an electrophoretic mobility. The association between AZGP1 and tumorigenesis was initially explored in prostate and breast cancer tissues [18,22]. Meanwhile, the AZGP1 diagnostic value in carcinoma effusion or serum of prostate and breast cancer patients has been reported in some studies [20,23,24]. Agesen *et al*. [25] using exon-level microarrays in a multi-medical center, multi-ethnic and large-scale sample study, found high *AZGP1* gene expression at the transcriptional level in colorectal cancer. We evaluated AZGP1 mRNA and protein expression in fresh colon cancer tissues by qPCR and WB. The expression levels of AZGP1 mRNA and protein in colon cancer tissues were higher than those in matched normal mucosa, suggesting that AZGP1 was upregulated at the transcriptional and posttranscriptional level. These results suggested that AZGP1 might be a novel molecular marker and of significance in the diagnosis of colon cancer. ROC curve analyses showed that the AUC of AZGP1 was in between the AUCs of CEA and CA19-9, thus the predictive diagnostic accuracy of AZGP1 was in between CEA and CA19-9. The combination of AZGP1 with traditional serum biomarkers, CEA and CA19-9, resulted in an AUC greater than the individual ones, suggesting that the combination of traditional serum biomarkers and AZGP1 could provide a more accurate diagnostic result. All of the above results indicated that AZGP1 could be used as an effective diagnostic marker for colon cancer.

In order to further validate AZGP1 diagnostic value, we verified the above conclusion in TMA. Our data showed that AZGP1 expression level was significantly higher in tumor tissues than in normal tissues in a Chinese population which was consistent with the TCGA data derived from America population, suggesting that it could serve as a general diagnostic biomarker in different populations. We validated AZGP1 diagnostic value in serum by leave-one-out cross-validation, which showed that AZGP1 and the combination with traditional serum biomarkers were useful serum biomarkers of colon cancer in a Chinese population.

Further, we found that in tissue samples data and in serum samples data the AZGP1 expression level was positively correlated with AJCC clinical stage, the result indicating that colon cancer patients with higher AZGP1 expression levels were in significantly more advanced progression of the disease. Ji *et al*. [26] found that colorectal cancer patients with high expression of AZGP1 showed worse clinical outcomes. We propose that AZGP1 might play an important role in the process of malignant transformation in colon mucosa cells and/or in survival or maintenance of malignant transformed cancer cells. A more detailed mechanistic exploration of the *AZGP1* gene function will be the subject of a future study in the laboratory.

Taken together, our study is the first comprehensive evaluation of AZGP1 variation at the mRNA and protein level in human colon cancer. Our study has further explored the diagnosis value of AZGP1 by immunohistochemistry in TMA and by ELISA in serum. Our study demonstrated that AZGP1 is a potential serum marker of colon cancer that may be correlated with tumorigenesis. Our results might provide useful information for the development of novel biomarkers for the clinical diagnosis of colon cancer.

## 4. Experimental Section

### 4.1. Human Tissue Specimens and Patient Information

All patient-derived specimens and patients’ information were collected and archived under protocols approved by the institutional review board of Shanghai Jiao Tong University affiliated Shanghai First People’s Hospital Medical Center. A written informed consent was obtained from each subject before the trials.

For qPCR and WB analysis, tissue samples were gathered from 28 patients who had recently undergone colectomy. These 28 pairs of fresh tissue were subpackaged, immediately frozen in liquid nitrogen, and subsequently stored at −80 °C. 

The tissue samples for TMA construction, obtained from 190 patients with primary colon cancer who had undergone surgery at the Shanghai Jiao Tong University affiliated Shanghai First People’s Hospital Gastrointestinal Cancer Center, were fixed in formalin and then embedded in paraffin for immunohistochemical analysis. No chemotherapy or radiotherapy was administered to patients before surgery. The diagnosis was confirmed by at least two pathologists. Staging was based on pathological findings, according to the American Joint Committee on Cancer (AJCC). There were 82 male and 108 female patients with a mean age of 66 years (range, 22–95 years). The tissue microarray constructed using the specimens described above comprised of primary tumor tissue samples, corresponding normal tissue samples. According to AJCC, we classified 24 cases as stage I, 81 as stage II, 68 as stage III, and 17 as stage IV.

For ELISA, a total of 160 serum samples were collected from Shanghai Jiao Tong University affiliated Shanghai First People’s Hospital, including 120 patients with colon cancer and 40 health controls. The 120 patients serum samples were collected before surgical operation, and these patients did not receive radiotherapy or chemotherapy prior to the serum samples collection and did not suffer from other malignancies with a mean age of 66.8 years (range, 26–93 years), The 40 healthy controls were collected from a physical examination center with a mean age of 39.2 years (range, 23–59 years).

### 4.2. Quantitative Real-Time Polymerase Chain Reaction (qPCR)

RNA isolation was performed according to the manufacturer’s instructions (TRIzol, Invitrogen, Carlsbad, CA, USA). AZGP1 mRNA level was examined in 28 colon cancer tissues and paired normal tissues by qPCR analysis using the SYBR Green RT-PCR kit (TaKaRa, Dalian, China), according to the manufacturer’s instructions. AZGP1 mRNA was amplified using the sense primer, 5'-AGGGAAGGTTTGGTTGTGAGAT-3', and the antisense primer, 5'-GGCTGGGATTTCTTTGTTGAAT-3' (107-bp fragment). β-actin mRNA was amplified using the sense primer, 5'-CTGGGACGACATGGAGAAAA-3', and the antisense primer 5'-AAGGAAGGCTGGAAGAGTGC-3' (564-bp fragment). The PCR conditions were as follows: initial denaturation at 95 °C for 10 min, followed by 40 cycles of denaturation at 95 °C for 10 s, annealing at 58 °C for 15 s, and elongation at 72 °C for 1 min. The expression level of AZGP1 was normalized to that of β-actin. The difference in AZGP1 expression between cancer tissues and paired normal tissues was calculated using the 2^−ΔΔ*C*t^ method. AZGP1Δ*C*t = *C*t_AZGP1_ − *C*t_β-actin_; AZGP1ΔΔ*C*t = AZGP1Δ*C*t_tumor_ − AZGP1Δ*C*t_no-tumor_.

### 4.3. Western Blot Analysis

Total proteins were extracted from fresh colon tissue frozen at −80 °C using a Whole Protein Extraction Kit (Chemicon, Billerica, MA, USA) and the total-protein concentration was measured using the BCA protein assay kit. Equivalent amounts of protein from each sample were electrophoresed on a 10% SDS-polyacrylamide gel and then transferred to a polyvinylidene difluoride (PVDF) membrane. The membranes were blocked in 5% fat-free milk solution for 1 h at room temperature, and then cut into strips according to the protein markers before incubating them with a primary monoclonal antibody to AZGP1 (1:1000, Abcam, Cambridge, UK) or a primary monoclonal antibody to tubulin overnight at 4 °C. After being washed with Tris-buffered saline/tween 20 (TBST), the strips were incubated with a secondary antibody against mouse immunoglobulin G for 4 h at room temperature. The proteins on the strips were then detected using enhanced chemiluminescence (Pierce Biotechnology, Rockford, IL, USA) and exposure to X-ray film visualizer.

### 4.4. Enzyme-Linked Immunosorbent Assay

The serum concentrations of AZGP1, CEA and Carbohydrate 19-9 (CA19-9) were measured by commercially available AZGP1, CEA and CA19-9 ELISA kits (Abnova, Heidelberg, Germany) according to the manufacturer’s recommendations. The absorbent value was measured at 450 nm by microplate reader, and the concentration was calculated according the standard curve.

### 4.5. Immunohistochemistry

AZGP1 protein was detected by immunostaining of the tissue microarray, using a rabbit polyclonal antibody against human AZGP1 (1:200; Abcam, Cambridge, UK). The immunohistochemistry results were analyzed by two pathologists without disclosing the patient diagnosis to them. The results were expressed as the score of the staining intensity and extent. Staining intensity or AZGP1 was scored as 0 (negative), 1 (weak), and 2 (strong). Staining area was scored as 0 (0%), 1 (1%–25%), 2 (26%–50%), 3 (51%–75%), and 4 (76%–100%), according to the percentage of positive-stained cells. According to the sum of staining intensity and staining extent scores, the final immunohistochemistry results were classified into 3 groups: 0–1 = negative, 2–4 = weak positive and 5–6 = strong positive [27].

### 4.6. Statistical Analysis

For continuous variables, data were expressed as the median and inter-quartile range and compared using the Mann–Whitney *U*-test for two-group comparisons or the Kruskal–Wallis test for three groups. The relationship between AZGP1 expression in TMA and clinicopathological variables was analyzed by chi-square test. Receiver operating characteristic (ROC) curves were established to evaluate the diagnostic value of serum markers. The Youden index was used to select the cutoff value that determined the sensitivity and specificity. Leave-one-out cross-validation was used to validate the diagnostic value. *p* < 0.05 was considered to be statistically significant. Analyses were performed using the SPSS statistical software program version 13.0 (SPSS Inc., Chicago, IL, USA).

## 5. Conclusions

AZGP1 was significantly upregulated at the transcriptional and posttranscriptional level in colon cancer tissues. Additionally, high serum concentrations of AZGP1 were detected in colon cancer patients, and the combination of AZGP1 with traditional serum biomarkers, CEA and CA19-9, resulted in a more accurate diagnostic result than the individual ones. Our study demonstrated AZGP1 could be a novel molecular marker and of significance in the diagnosis of colon cancer in Chinese population.

## Supplementary Materials

Supplementary materials can be found at http://www.mdpi.com/1422-0067/16/01/0691/s1.
